# In silico drug combination discovery for personalized cancer therapy

**DOI:** 10.1186/s12918-018-0546-1

**Published:** 2018-03-19

**Authors:** Minji Jeon, Sunkyu Kim, Sungjoon Park, Heewon Lee, Jaewoo Kang

**Affiliations:** 1 Department of Computer Science and Engineering, Korea University, Seoul, Korea; 2 Interdisciplinary Graduate Program in Bioinformatics, Korea University, Seoul, Korea

**Keywords:** Combination therapy, in silico, Synergy prediction

## Abstract

**Background:**

Drug combination therapy, which is considered as an alternative to single drug therapy, can potentially reduce resistance and toxicity, and have synergistic efficacy. As drug combination therapies are widely used in the clinic for hypertension, asthma, and AIDS, they have also been proposed for the treatment of cancer. However, it is difficult to select and experimentally evaluate effective combinations because not only is the number of cancer drug combinations extremely large but also the effectiveness of drug combinations varies depending on the genetic variation of cancer patients. A computational approach that prioritizes the best drug combinations considering the genetic information of a cancer patient is necessary to reduce the search space.

**Results:**

We propose an in-silico method for personalized drug combination therapy discovery. We predict the synergy between two drugs and a cell line using genomic information, targets of drugs, and pharmacological information. We calculate and predict the synergy scores of 583 drug combinations for 31 cancer cell lines. For feature dimension reduction, we select the mutations or expression levels of the genes in cancer-related pathways. We also used various machine learning models. Extremely Randomized Trees (ERT), a tree-based ensemble model, achieved the best performance in the synergy score prediction regression task. The correlation coefficient between the synergy scores predicted by ERT and the actual observations is 0.738. To compare with an existing drug combination synergy classification model, we reformulate the problem as a binary classification problem by thresholding the synergy scores. ERT achieved an F1 score of 0.954 when synergy scores of 20 and -20 were used as the threshold, which is 8.7% higher than that obtained by the state-of-the-art baseline model. Moreover, the model correctly predicts the most synergistic combination, from approximately 100 candidate drug combinations, as the top choice for 15 out of the 31 cell lines. For 28 out of the 31 cell lines, the model predicts the most synergistic combination in the top 10 of approximately 100 candidate drug combinations. Finally, we analyze the results, generate synergistic rules using the features, and validate the rules through the literature survey.

**Conclusion:**

Using various types of genomic information of cancer cell lines, targets of drugs, and pharmacological information, a drug combination synergy prediction pipeline is proposed. The pipeline regresses the synergy level between two drugs and a cell line as well as classifies if there exists synergy or antagonism between them. Discovering new drug combinations by our pipeline may improve personalized cancer therapy.

## Background

Researchers have been working for years to develop new cancer drugs with “one drug, one target, one disease” strategy. However, the discovery and approval of new molecular entities has been on the decline in the pharmaceutical industry [1]. According to the Biotechnology Innovation Organization’s 2016 report, the success rate of new drug development in oncology between 2006 and 2015 was 5.1%, the lowest among 14 major diseases while the overall average was 9.6% [2]. Cancer is a complex disease in which various molecules interact; the networks formed by interactions are robust, redundant, and compensatory [3]. When cancer is treated with a single drug that targets a specific molecule, the cancer tries to discover bypasses in the networks and finds alternative cancer addicted pathways to evade apoptosis or proliferate [4]. Thus, it is difficult to develop a sustainable targeted cancer drug.

Drug combination therapy, which is considered as an alternative to single drug therapy, can potentially reduce resistance and toxicity, and have synergistic effects [5]. As drug combination therapies are widely used in clinics for hypertension, asthma, and AIDS, they have also been proposed for treating cancer. Since MOPP(Mechlorethamine, Oncovin, Procarbazine, Prednisone) has been used for Hodgkin’s lymphoma in the 1960s, more than 50 drug combination therapies have been approved by the FDA. For example, the combination of vemurafenib, which targets BRAF, and cobimetinib, which targets MAP2K1, was approved by the FDA in 2015 for use in treating BRAF mutated melanoma.

However, it is difficult to select and experimentally evaluate effective combinations because the number of cancer drug combinations is extremely large. The number of FDA-approved cancer drugs is more than 200. Combining two drugs will result in at least 19,900 combinations. Since several thousands of chemical compounds are used in clinical trials, the number of combinations to be tested will be in the millions. If we are combining three or more drugs, the number will increase exponentially.

Moreover, it is assumed that drug combination therapy will always be better than a single drug treatment, but it may have synergistic or antagonistic effects depending on the genetic variation of a person [6]. Therefore, it is necessary to consider the genetic characteristics of a patient when using drug combination therapy. Genetic variation makes it more difficult to generate biological hypotheses and experimentally discover effective drug combinations.

For this reason, an efficient computational approach that prioritizes the best synergistic drug combinations while considering a cancer patient’s genetic information is necessary. Many computational methods have been developed to find effective personalized drug combination therapies. As this problem is important, a community based-competition called the DREAM Challenge was launched to help generate drug combination prediction models [7]. Although various models have been proposed for the DREAM Challenge, the models are insufficient for making a scalable prediction system for various cell lines and drug combinations using drug screening data of 14 drugs on the OCI-LY3 DLBCL cell line.

There are various machine learning-based approaches. A sensitivity-based approach [8] uses drug-response data on BRAF mutant melanoma cancer cell lines and employs a classification model that predicts synergistic drug combinations. Since this classification model uses only the BRAF mutant melanoma cell lines, it is difficult to generalize to all cancer types. It also cannot rank synergistic drug combinations. Drug target- or kinase inhibition profile-based approaches [9, 10] find potential combinational targets based on individual drug effects. Matlock et al. attempted to find effective and less toxic combinational targets using drug sensitivity data of cancer cell lines and normal cells. The method by Matlock et al. is based on calculating the effectiveness probability of combinational targets using a drug’s effect on the cell and the target information of the drug. However, to employ these approaches, we should know the effects of drugs including unintended off-target effects that are difficult to obtain. Moreover, there may not exist any drug known to target the combinational targets suggested by these systems. Network-based modeling [11–13] uses cancer pathways or drug-target networks for discovering drug combinations. Unlike the other models, network-based models can be utilized to hypothesize intervention mechanisms because the models are based on prior knowledge such as molecular interaction networks and drug-target networks. However, the accuracy of the models can be lowered by the existing networks’ limited coverage. Although many studies have been proposed to find effective drug combinations, the studies are either limited to specific cancer types [8], require comprehensive off-target information [9, 10], or are based on incomplete prior knowledge [11–13], making it difficult to perform scalable computational drug combination prediction using high-throughput drug screening data.

To address these problems, we propose an in-silico method that uses unbiased high-throughput drug screening data for personalized synergistic drug combination therapy discovery for the treatment of cancer. Our machine learning models use the genomic information of cancer cell lines from various cancer types, regress the synergy level between two drugs and a cell line as well as classify whether there exists synergism or antagonism between them, and make synergistic rules for predicting the synergism of drug combinations using the features. The source code will be available at http://infos.korea.ac.kr/dcpipe/.

## Methods

### Approach

**Fig. 1:**
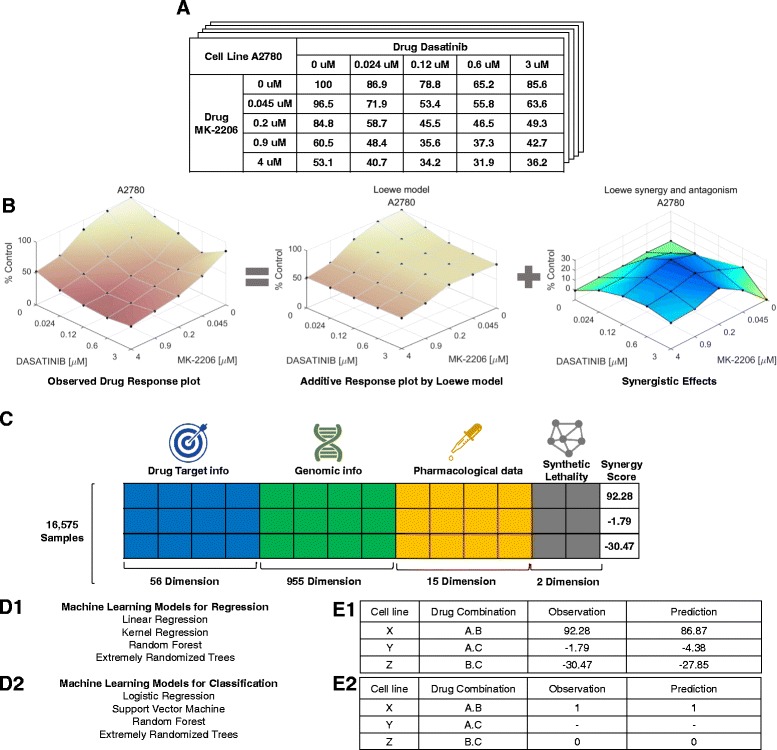
**a** Reformatted experimental data from O‘Neil’s dataset for analyzing by Combenefit. **b** Synergy scores calculated by Combenefit. **c** For predicting synergy scores, each sample is vectorized. The vector contains drug targets, genomic information of a cell line, pharmacological data, and other external knowledge such as synthetic lethality. **d** and **e** Predicted synergy scores calculated using various machine learning models. Pearson correlation coefficient and F1 score were used as the evaluation metrics for the regression models and classification models, respectively

We constructed a personalized drug combination synergy prediction pipeline and Fig. 1 shows how our pipeline works with an example data. Synergy in the pipeline is defined as a synergy score quantified using a tool called Combenefit [14]. We used genomic information such as gene expression, mutation, and copy number variation data of cell lines from various unbiased cancer types, and target information of cancer drugs to predict synergy scores. In addition, single drug response data and synthetic lethality data are used as well.

The high dimensionality problem of the feature space was the main difficulty when constructing a model to predict synergism. The number of genomic features such as mutations and expressions of more than 10,000 genes should be reduced to a much smaller size. To address this, we reduce the feature dimensions by using only the mutations or expression levels of the genes in cancer-related pathways. Finally, the features for each sample are represented as a 1,028-dimensional vector.

We use various machine learning models including linear regression, RBF(Radial Basis Function) kernel-based ridge regression, random forest, and so on and compare them. We analyze the results and make synergistic rules for predicting the synergism of drug combinations using the features.

### Datasets

We use O’Neil’s high-throughput drug combination screening data [15]. This data contains 23,052 experimental results for 583 drug combinations and 39 cancer cell lines. Each experiment consists of a 5 by 5 matrix of drug concentrations. Unlike other high-throughput screening data, this data includes unbiased cell lines from various sites, such as breast, lung, and colon. Drugs are also unbiased; for example, some drugs are FDA-approved, chemical compounds used in clinical trials, chemotherapy drugs, or targeted therapy drugs. We used only 31 cell lines whose genomic profiles are publicly available and only 16,575 experimental data samples excluding duplicates.

For evaluating machine learning models, whole samples were randomly divided into a training set and a test set at a ratio of 80:20. The samples in the training set were randomly divided into five equal-sized subsamples and used as 5-fold cross-validation sets. The cross-validation sets are used to decide hyperparameters for machine learning models.

### Synergy score calculation

**Fig. 2:**
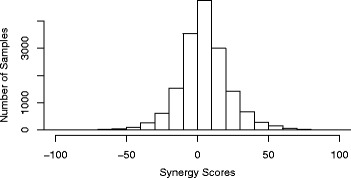
Synergy Score Distribution. The average synergy score of 16,575 samples is 4.52 and the standard deviation is 20.65

We employed a tool called Combenefit [14] to quantify the synergy level between two drugs and a cancer cell line using dose-response data. Combenefit enables model-based quantification of drug combinations by comparing additive and actual effect for given dose-response data as shown in Fig. 1b. This tool calculates the difference between the Loewe model-based expected additive effect and the actual effect of the drug combination. We call this difference value a synergy score. If the actual effect of a drug combination is greater than the additive effect, the synergy score is greater than zero. Otherwise, it is less than zero. A higher synergy score denotes greater synergy of the corresponding drug combination. As shown in Fig. 2, the average synergy score of 16,575 samples is about 4.52 and the standard deviation is 20.65. The synergy scores obtained using Combenefit are target variables in the regression model in our pipeline. The regression model that predicts the synergy scores is the drug combination synergy prediction model.

### Features

A sample consisting of a cell line and two drugs is represented as a feature vector. The vector contains genomic information of the cell line and target information of the drugs. Other information such as pharmacological experimental results of the cell line and the drugs is also included in the vector. A detailed description of the features follows.

#### Genomics

Information of gene mutations, gene expression, and copy number variations for 31 cell lines among 39 cell lines in the dataset from O‘Neil was obtained from COSMIC [16]. A high dimensionality problem exists because of the large number of genes in the dataset. Therefore, we select genes in cancer-related pathways. cBioPortal [17] defines 14 gene sets consisting of cancer-related pathways such as DNA damage response or RTK signaling pathways. We use only the mutations, expression levels, and copy number variations of the genes in the gene sets. For mutations, if there is an amino acid variation in the gene, the variation is marked as 1 in the feature vector. The normalized expression levels are marked in the feature vector. For copy number variations, each gene is divided into two features: amplification and deletion. Amplification and deletion are marked as binary.

#### Targets

A total number of 56 drug targets of 38 drugs in the dataset were obtained from DrugBank [18] and GDSC [19]. Targets of two drugs in a sample are marked as 1.

#### Mono-therapy

Mono-therapy information about drug effects on cell lines is used. IC50 is the half maximal inhibitory concentration. Drug Sensitivity Score (DSS) [20] calculates the area under the IC50 plot. Einf is the effect observed when an infinite amount of a drug used. We use the sum and difference of these values of two drugs.

#### Addition score

The addition score indicates the additive effects between two drugs using only the mono-therapy response data of the two drugs (excluding the actual observations of combination responses). We calculate the addition score by comparing the Loewe additive effect of the two drugs with the non-drug effect as shown in the additive response plot in Fig. 1b. Addition scores were also calculated using Combenefit.

#### Mean/median synergy scores of similar samples

We assumed that the average synergy score of the samples in the training set containing the same cell line or the same drug combination as the test sample would be helpful for predicting the synergy score of a sample in the test set. For example, we hypothesize that the average synergy score of the samples in the training set that contains the cell line HT29 would be helpful for determining whether the drug combination AZD1775 and MK-8776, and the cell line HT29 in the test set are synergistic. Likewise, we hypothesize that for the same test sample, the average synergy score of the training samples containing AZD1775 and MK-8776 would be helpful for predicting the synergism of the test sample. To capture this, we sub-grouped the samples in the training set by cell line or drug combination, and used the mean and median values of their synergy scores as features of the given sample. For the sample with the drug combination AZD1775 and MK-8776, and the cell line HT29, the mean and median synergy scores of the following samples are obtained: samples with HT29, samples with either AZD1775 or MK-8776 (i.e., sharing at least one drug with the given sample), samples with both AZD1775 and MK-8776, samples with either AZD1775 or MK-8776, and HT29 (i.e., sharing at least one drug and the cell line). This is intended to capture some inherent pharmacological similarities among drugs and cell lines.

#### Synthetic lethality

Synthetic lethality occurs when perturbations such as mutations, malfunctions, or inhibitions of two genes lead to cell death. SynLethDB [21] is a comprehensive database of over 20,000 synthetic lethality gene pairs from various types of sources such as biomedical literature and databases, and provides the confidence scores of the pairs. We use this information to determine how many synthetic lethality pairs occur in a sample. For each sample, we sum the confidence scores of the pairs when one of the two genes is perturbed and the rest of the gene is targeted by drugs.

## Results and discussion

We evaluate the machine learning models on both the regression and classification tasks. In the regression task, the models are trained to predict the synergy scores of samples, and in the classification task, the models are trained to classify samples into the following two groups: synergism or antagonism.

### Regression

We use the Pearson correlation coefficient to evaluate the performance of all the machine learning models used for our pipeline. We have trained a variety of machine learning models. For the hyperparameter optimization of each model, we use a grid search to find hyperparameters that maximize the 5-fold cross-validation performance. For example, for a regularized coefficient for a kernel-based ridge regression model or a C value for an SVM model, we performed a grid search over exponentially increasing values from 0.0001 to 1 or 0.0001 to 100. Then each model is trained on the training set with the specified hyperparameters and evaluated on the test set for performance measurement.

We use linear and nonlinear machine learning models. The models are implemented using scikit-learn [22] which is a machine learning package in Python. Table 1 shows the correlation coefficient between the observed values and predicted values of each model. Overall, the performance of linear models is clearly lower than that of nonlinear models; however, the performance difference between nonlinear models is small. The Extremely Randomized Trees (ERT) model [23], which is a tree-based ensemble model, obtains the highest correlation coefficient of 0.738.

**Table 1:** Prediction Evaluation (correlation coefficient)

	Model	Correlation coefficient	Correlation coefficient	Correlation coefficient	Correlation coefficient
			(1700 samples with >10 or <-10)	(1177 samples with >15 or <-15)	(780 samples with >20 or <-20)
Linear	Elastic net	0.65	0.696	0.711	0.733
	Ridge regression	0.661	0.717	0.742	0.752
Nonlinear	Kernel ridge regression (RBF)	0.728	0.773	0.795	0.822
	Random forest	0.731	*0.792*	0.8	*0.827*
	Extremely randomized trees	*0.738*	0.785	*0.816*	0.821

Italicized values are the best performance for each experiment

ERT model’s basic structure is similar to that of Random Forest in which every tree uses a randomly selected subset of samples as well as a randomly selected subset of features, whereas a tree in ERT model selects a random feature from the subset of features rather than the most discriminant feature when branching. We believe that the ERT model outperforms the others for the following reasons: ERT model’s extreme randomness introduced by the random subsampling of samples and features, and the random feature selection during the branching. This randomness helps reduce the variance of the model. The reduced variance, in turn, makes the model more robust in dealing with noisy data such as our high-throughput experimental drug screening data.

In addition, we collected only samples whose synergy were predicted to be very small (e.g., < -10) or large (e.g., >10). We computed the performance of the models only for the samples and reported the results in Table 1 (column 3 to 5). The correlation coefficient of the samples that are predicted to be greater than 20 or less than -20 by Random Forest is 0.827. All the models achieve better performance on the samples with larger or smaller predicted values, which means that our models are suitable for predicting synergism or antagonism.

**Fig. 3:**
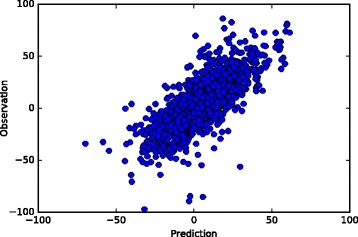
Observation and prediction values obtained by the Extremely Randomized Trees model (correlation coefficient is 0.738)

Figure 3 shows a plot of the observed synergy scores and the predicted synergy scores computed by the Extremely Randomized Trees model. In addition, Table 2 shows the correlation coefficient for each cell line computed by the model. The most predictable cell line is RPMI7951 and the least predictable cell line is NCIH23. Column 4 in Table 2 shows the most synergistic drug combination for each cell line in the test set. The most synergistic drug combinations are computed from real observations in O’Neil’s data. Column 5 shows the rank of the combination predicted by our model. Our model correctly predicts the most synergistic combination, from approximately 100 candidate drug combinations, as the top choice for 15 out of the 31 cell lines. For 28 out of the 31 cell lines, our model predicts the most synergistic combination in the top 10 among approximately 100 drug combinations.

**Table 2:** Correlation coefficients of 31 cell lines calculated by the Extremely Randomized Trees model

Cell line name	Site	Correlation coefficient	Best observed	Its predicted rank out of
			drug combination	N drug combinations
RPMI7951	Skin	0.893	MK-4827.TEMOZOLOMIDE	1 out of 88
HCT116	Colon	0.892	ETOPOSIDE.MK-2206	7 out of 105
HT29	Colon	0.889	AZD1775.MK-8776	2 out of 116
SW620	Colon	0.874	BEZ-235.MK-8669	1 out of 108
PA1	Ovarian	0.868	MK-2206.SUNITINIB	5 out of 85
A2780	Ovarian	0.865	DASATINIB.MK-5108	1 out of 112
MDAMB436	Breast	0.864	MK-4827.TEMOZOLOMIDE	1 out of 114
VCAP	Prostate	0.856	BEZ-235.MK-8669	1 out of 109
LOVO	Colon	0.839	MK-4827.TEMOZOLOMIDE	1 out of 121
OV90	Ovarian	0.833	AZD1775.MK-8776	1 out of 99
OCUBM	Breast	0.831	AZD1775.MK-8776	1 out of 62
NCIH1650	Lung	0.83	DASATINIB.MK-2206	2 out of 91
A2058	Skin	0.829	MK-4827.MK-8776	28 out of 119
SKMES1	Lung	0.825	BEZ-235.MK-8669	1 out of 113
A427	Lung	0.807	MK-8776.TEMOZOLOMIDE	9 out of 105
SKOV3	Ovarian	0.798	ERLOTINIB.MK-8669	3 out of 105
UACC62	Skin	0.797	GEMCITABINE.MK-8776	5 out of 120
DLD1	Colon	0.79	MK-2206.MK-8669	2 out of 116
ES2	Ovarian	0.789	BEZ-235.MK-8669	1 out of 125
NCIH2122	Lung	0.766	DEXAMETHASONE.MK-2206	8 out of 137
SKMEL30	Skin	0.709	BEZ-235.MK-8669	1 out of 105
A375	Skin	0.691	MK-4827.TEMOZOLOMIDE	1 out of 103
RKO	Colon	0.688	GELDANAMYCIN.PD325901	20 out of 117
HT144	Skin	0.669	MK-2206.SN-38	7 out of 124
NCIH520	Lung	0.643	DASATINIB.TOPOTECAN	20 out of 124
UWB1289	Ovarian	0.63	BEZ-235.MK-8669	1 out of 125
MSTO	Lung	0.628	BEZ-235.MK-8669	1 out of 86
SW837	Colon	0.626	BEZ-235.DASATINIB	8 out of 110
OVCAR3	Ovarian	0.554	AZD1775.SN-38	5 out of 93
T47D	Breast	0.493	DOXORUBICIN.MK-8669	1 out of 61
NCIH23	Lung	0.478	L778123.MK-8669	2 out of 117

### Classification

To compare our model with an existing drug combination synergy classification model, we reformulate the problem as a binary classification problem by thresholding the synergy scores. If a synergy score is greater than the upper threshold, it is classified as a positive class. If the value is less than the lower threshold, it is classified as a negative class; otherwise, it is dropped. After generating classification models using the classified samples, the F1 score is used to evaluate the models. F1 score is defined as the harmonic mean of precision and recall, i.e., F1 = 2 ×*precision*×recall/(precision+recall), where precision=true positives/(true positives + false positives) and recall=true positives/(true positives+false negatives). F1 score is one of the standard metrics for evaluating a classifier’s performance. F1 score is commonly used when the target classes are imbalanced. In our dataset, 10,392 samples have synergy scores of greater than 0 (positive class) and 6,183 samples have synergy scores of less than 0 (negative class). Therefore, we used the F1 score to evaluate the performance of the classification models.

Table 3 shows the F1 score of each model by thresholding the synergy scores. The Extremely Randomized Trees model achieves the highest F1 score of 0.954 with a threshold of ±20. As the threshold increases, performance tends to improve. The Extremely Randomized Trees model achieves the best performance for the thresholds of 0, ±15, and ±20. Compared with the baseline model [8], our pipeline based on the Extremely Randomized Trees model improves the performance by 8.7%.

**Table 3:** Prediction evaluation results (F1 score)

Model	F1 (threshold=0)	F1 (threshold= ±10)	F1 (threshold= ±15)	F1 (threshold= ±20)
Baseline [8]	0.784	0.856	0.871	0.877
Logistic regression	0.844	0.908	0.912	0.923
Support vector machine	0.84	0.909	0.915	0.917
Random forest	0.86	*0.934*	0.939	0.944
Extremely randomized trees	*0.865*	0.933	*0.948*	*0.954*

Italicized values are the best performance for each experiment

### Synergistic rule generation: a qualitative analysis

In addition to the quantitative analysis of our pipeline’s performance, we also conducted a qualitative evaluation. For the qualitative evaluation, we use combinations of feature conditions and construct rules to predict synergism. Although the Extremely Randomized Trees model achieves the best performance, it randomly selects features when it splits, which makes it difficult to interpret the results. For this reason, we choose the Random Forest model because its results are easy to interpret. We extract frequent decision paths generated by the Random Forest model and inspect the paths for predicting synergism. We generate four potential synergistic rules from the paths and report them in Table 4.

**Table 4:** Synergistic rules

Rule	Positive	Negative	*p*-value	Reference
	synergy score	synergy score		
Target: mTOR, DNA GEX: underexpressed HES5	23.055	8.651	1.3*e*^−10^	[24, 25]
Target: EGFR, mTOR, PI3K GEX: underexpressed SNW1	22.219	8.588	6*e*^−5^	
Target: mTOR, PI3K, DNA GEX: underexpressed MAPK14, underexpressed NOTCH4	19.8	6.231	5*e*^−7^	
Target: SRC, TOP1 GEX: underexpressed TGFBR2, underexpressed ERBB3	28.089	7.113	2*e*^−5^	

The most significant synergistic rule is about that a chemotherapy drug(represented as DNA) combined with an mTOR-targeting drug is synergistic when HES5 is underexpressed. The Random Forest model predicts samples that satisfy these constraints as synergy. The average synergy score of the positive samples (i.e., mTOR inhibitor + chemotherapy for cells with HES5 underexpression) is 23.055. Conversely, the average synergy score of the negative samples (i.e., mTOR inhibitor + chemotherapy for cells with no HES5 underexpression) is 8.651 which is 14.4 lower than the score of the positive samples (*p*-value= 1.3*e*^−10^). Down-regulation of HES5 indicates inactivated Notch signaling and results in sensitive response to a chemotherapy drug [24]. Inactivated Notch signaling is also involved in the down-regulation of mTOR [25]. Thus, it seems that the mTOR inhibitor and underexpressed HES5 enhance the efficacy of chemotherapy by double-blocking mTOR.

In Table 4, we report three more potential synergistic rules which we could not find supporting evidence from the literature. These rules can become hypotheses and can be verified by wet lab experiments. If validated, these synergistic rules would be used as potential biomarkers for new drug combination therapy. This suggests that our pipeline can generate hypotheses in a completely data-driven way. As more drug screening data accumulates, our pipeline can automatically find more plausible hypotheses for drug combination biomarkers.

## Conclusion

Synergistic drug combinations are different for each cell line. Therefore, we proposed a computational pipeline for personalized synergistic drug combination therapy discovery using unbiased high-throughput drug screening data. We generated a 1,028-dimensional feature vector to represent a sample’s genomic information such as mutations and expressions of genes in cancer-related pathways, and pharmacological information. The tree-based ensemble models achieved the best regression and classification performance. The correlation coefficient for the regression was 0.738 and the F1 score for the classification was 0.954. We demonstrated that our proposed model can be applied to patient samples in place of cells when it is employed in a clinical setting. We showed that our model can predict and prioritize the best drug combinations for patients using their genomic information. Finally, we generated synergistic rules from the frequent paths of the Random Forest model and validated the rules by literature survey. With further experiments, the discovered rules can be tested for biomarkers of drug combination therapies.
